# Implementing Horizon Scanning as a tool for the strategic development of regulatory guidelines for nanotechnology-enabled health products

**DOI:** 10.3389/fmed.2023.1308047

**Published:** 2024-01-11

**Authors:** Francisco D. Rodríguez-Gómez, Dominique Monferrer, Oriol Penon, Pilar Rivera-Gil

**Affiliations:** ^1^Asphalion SL, Barcelona, Spain; ^2^Integrative Biomedical Materials and Nanomedicine Lab, Department of Medicine and Life Sciences, Universitat Pompeu Fabra, Barcelona Biomedicine Research Park (PRBB), Doctor Aiguader, Barcelona, Spain

**Keywords:** regulatory science, horizon scanning, nanomedicine, nanotechnology-enabled health product, regulatory guideline

## Abstract

Strategic regulatory development is essential to ensure that new innovations in nanotechnology-enabled health products (NHPs) successfully reach the market and benefit patients. Currently, the lack of specific regulatory guidelines for NHPs is considered one of the primary causes of the so-called “valley of death” in these products, impacting both current and future advancements. In this study, we have implemented a methodology to anticipate key trends in NHP development and compare them with the current regulatory landscape applicable to NHPs. This methodology relies on Horizon Scanning, a tool commonly used by policymakers to foresee future needs and proactively shape a regulatory framework tailored to those needs. Through the application of this methodology, different trends in NHP have been identified, notably NHPs for drug delivery and dental applications. Furthermore, the most disruptive elements involve NHPs that are multicomposite and multifunctional, harnessing nano-scale properties to combine therapeutic and diagnostic purposes within a single product. When compared with the regulatory landscape, current regulations are gradually adapting to accommodate emerging trends, with specific guidelines being developed. However, for the most disruptive elements, multicomposite and multifunctional NHPs, their novelty still poses significant regulatory challenges, requiring a strategic development of guidelines by regulatory agencies to ensure their safe and effective integration into healthcare practices. This study underscores the importance of proactive regulatory planning to bridge the gap between NHP innovation and market implementation.

## 1 Introduction

The global nanotechnology market is expected to see a significant growth throughout the current decade (1). Predictions suggest a compound annual growth rate (CAGR)^1^ ranging from 9.2% to 36.4% between 2000 and 2030. The global nanomaterials market was valued at USD 7.1 billion in 2020, and it is predicted to grow to USD 13.60 billion by 2027. This trend is largely driven by their increasing incorporation into nanotechnology-enabled health products (NHPs),^2^ particularly in applications for drug delivery. Namely, the fastest growth is expected to occur in the Asia-Pacific region (2).

However, the successful innovation of NHPs must be supported by the development of a robust regulatory framework to ensure that the quality, safety, and efficacy of these products can be adequately assessed by health authorities. In 2020, the European Medicines Agency (EMA) acknowledged the need to “develop understanding of, and regulatory response to, nanotechnology and new materials in pharmaceuticals” (3). Among different underlying actions, the development and standardisation of novel testing methodologies for nanomedicines were identified as paramount, particularly for safety and quality assessments.

In line with this, the European Technology Platform for Nanomedicine’s 2016 report on the “Strategic Research and Innovation Agenda for Nanomedicine 2016–2030” emphasised early engagement with regulators about nanomedical trends to ensure adaptable regulations and a “fast but safe track” to innovation (4).

Despite these initiatives, the pace of regulations advancement is lagging behind that of NHP innovation (5). This discrepancy is frequently identified as a contributing factor to the “valley of death” in NHPs, a term used to describe the significant gap between the number of applications in research and development and the number of products (both medicinal products and medical devices) on the market (6, 7).

To prevent this situation, policy makers can utilize various tools to anticipate and address future regulatory needs. One such tool is Horizon Scanning, which is a systematic methodology designed to identify early signs of significant developments, opportunities and threats that could impact a specific area (8). The use of Horizon Scanning methodology is well-established among legislators. In health politics for instance, it is used by the governments of United Kingdom, the Netherlands and Singapore. In addition, international organizations like the World Health Organization (WHO) (9) and regulatory bodies such as the EMA, the British Medicines and Healthcare Products Regulatory Agency (MHRA) and the Japanese Pharmaceuticals and Medical Devices Agency (PMDA) also use Horizon Scanning-based methodologies for strategic regulatory development (10–13).

Horizon Scanning can be commonly implemented through two distinct approaches: exploratory scanning and issue-centred scanning. Exploratory scanning is a heuristic search for information aimed at identifying potential issues and signs by reviewing various sources of information or “scans” (see Table 1 for key terms used in Horizon Scanning). Conversely, issue-centred scanning begins with the analysis of a set of core documents to identify signals within a specific field of study (15). Although there is no standardised description for implementing the Horizon Scanning methodology (17), it is traditionally described as a process that includes the following steps: signal detection, filtration, prioritization, assessment, and dissemination (18). It is important to note that Horizon Scanning is not a simple prediction tool; predictions usually deal with broader topics and have longer-term impacts. In contrast, Horizon Scanning focuses more narrowly on specific topics in the short to medium term (18).

**Table 1:** Key terminology in Horizon Scanning.

Term	Definition
Weak signal	Early indicators of a potential change (10). They represent the first signs of paradigm shifts and future trends (14).
Wild card	Disruptive events of surprising character, low probability and a high impact (15).
Scan	Source document that may describe a weak signal or a wild card (16).

Again, for regulatory agencies, the application of future-oriented methodologies, such as Horizon Scanning, serves to pre-emptively identify upcoming needs. This foresight facilitates the creation of specific guidelines or legislation, ensuring that all innovations are well addressed within their applicable regulatory framework. The objective is to establish expertise in advance of evaluating forthcoming innovative products, thereby streamlining their path to market by minimizing potential developmental, legal or regulatory bottlenecks (12).

As previously discussed, the current regulatory state of the art applicable to NHPs, understood as the most recent versions of regulatory guidelines and technical standards published and under development (19), is a known cause of the “valley of death” for this kind of products. This situation is considered a challenge that could potentially escalate with the swift progression of nanotechnology. In this paper, we suggest utilizing the Horizon Scanning methodology to focus on the advancements in NHPs. The aim is to predict upcoming trends and improve the strategic development of regulatory guidelines.

## 2 Materials and methods

### 2.1 Regulatory database

Guidelines and other regulatory documents applicable to NHPs have been gathered in a regulatory database that includes references identified up to August 31st 2023 from the following sources:

- Competent authorities:

o European Medicines Agency (EMA): https://www.ema.europa.eu/en/human-regulatory/research-development/scientific-guidelines/multidisciplinary/multidisciplinary-nanomedicines.
o United States Food and Drug Administration (FDA): https://www.fda.gov/science-research/nanotechnology-programs-fda/nanotechnology-guidance-documents.

- European Commission:

o EU Science Hub: https://joint-research-centre.ec.europa.eu/scientific-activities-z/nanotechnology_en.
o Scientific Committee on Emerging and Newly Identified Health Risks (SCENHIR): https://health.ec.europa.eu/scientific-committees/former-scientific-committees/scientific-committee-emerging-and-newly-identified-health-risks-scenihr_en.

- Organization for the Economic Co-operation and Development (OECD), Safety of manufactured nanomaterials: https://www.oecd.org/science/nanosafety/.

- Standards emitting organizations:

o International Organization for Standardization (ISO), Technical committee ISO/TC 229 on Nanotechnologies: https://www.iso.org/committee/381983.html.
o American Society for Testing and Materials (ASTM), Technical subcommittee E56.08 on Nano-enabled Medicinal Products: https://www.astm.org/get-involved/technical-committees/committee-e56/subcommittee-e56.

### 2.2 Horizon Scanning methodology

The adaptation and implementation of the Horizon Scanning methodology has been developed as part of the objectives of this manuscript. It is applied in four stages based on exploratory scanning approach: (i) signal detection, (ii) filtration, (iii) prioritization, and (iv) assessment (18).

#### 2.2.1 Signal detection

Various scanners were used for signal detection such as scientific publication databases, clinical study databases, and international patent registries that encompass NHPs at different developmental stages. To effectively handle the volume of information, searches were performed during three distinct periods of time, ranging from January 2020 to June 2022 (refer to Table 2). Any results not directly relevant to a specific NHP were discarded.

**Table 2:** Information sources considered for Horizon Scanning.

Scan	String	Date		
		Scan period 1	Scan period 2	Scan period 3
Scientific publications – Scopus	(nanomaterial OR nanotechnology) AND (*medicine OR device) AND (treatment OR therapy OR prophylaxis OR diagnos*) AND health* Keywords must appear within title/abstract/keyworks Language: English Limit to document type: article	01/01/2020–22/06/2021	23/06/2021–13/01/2022	14/01/2022–15/06/2022
Patents register – European Patent Office (EPO)	EN_TI:(nano*) AND CPC2:(A61)	01/01/2020–10/08/2021	11/08/2021–13/01/2022	14/01/2022–15/06/2022
Clinical studies – Clinicaltrials.gov	Other terms: nano Status: exclude suspended or withdrawn	not defined–21/07/2021	22/07/2021–13/01/2022	14/01/2022–15/06/2022

Cooperative Patent Classification (CPC) code A61 is applicable to “medical or veterinary science; hygiene.” The symbol “*” is used for truncation of search terms. No lower time limit was set for clinical studies to maximize the inclusion of ongoing studies in the search results.

#### 2.2.2 Filtration and prioritization

Novelty was employed as a filtration criterion to eliminate irrelevant signals and as a prioritization standard for categorizing results into potential trends (weak signals) or disruptive elements (wild cards). Besides, the methodology for novelty quantification was adapted based on the novelty metrics proposed by Shah et al. (20). This widely recognised approach, originating from the field of design creativity research, characterises novelty as a measure of how unusual or unexpected an idea is compared to other ideas (20, 21).

#### 2.2.3 Assessment

The detected trends and wild cards were assessed for their potential impact on the current regulatory state-of-the art (i.e., guidelines and standards). This assessment involved a detailed analysis of how these detected signals fit within the scope of the existing regulatory science, as reflected in the regulatory database. The aim of this assessment was to identify potential areas of insufficient regulation.

### 2.3 Classification system for nanotechnology-enabled health products

**Figure 1:**
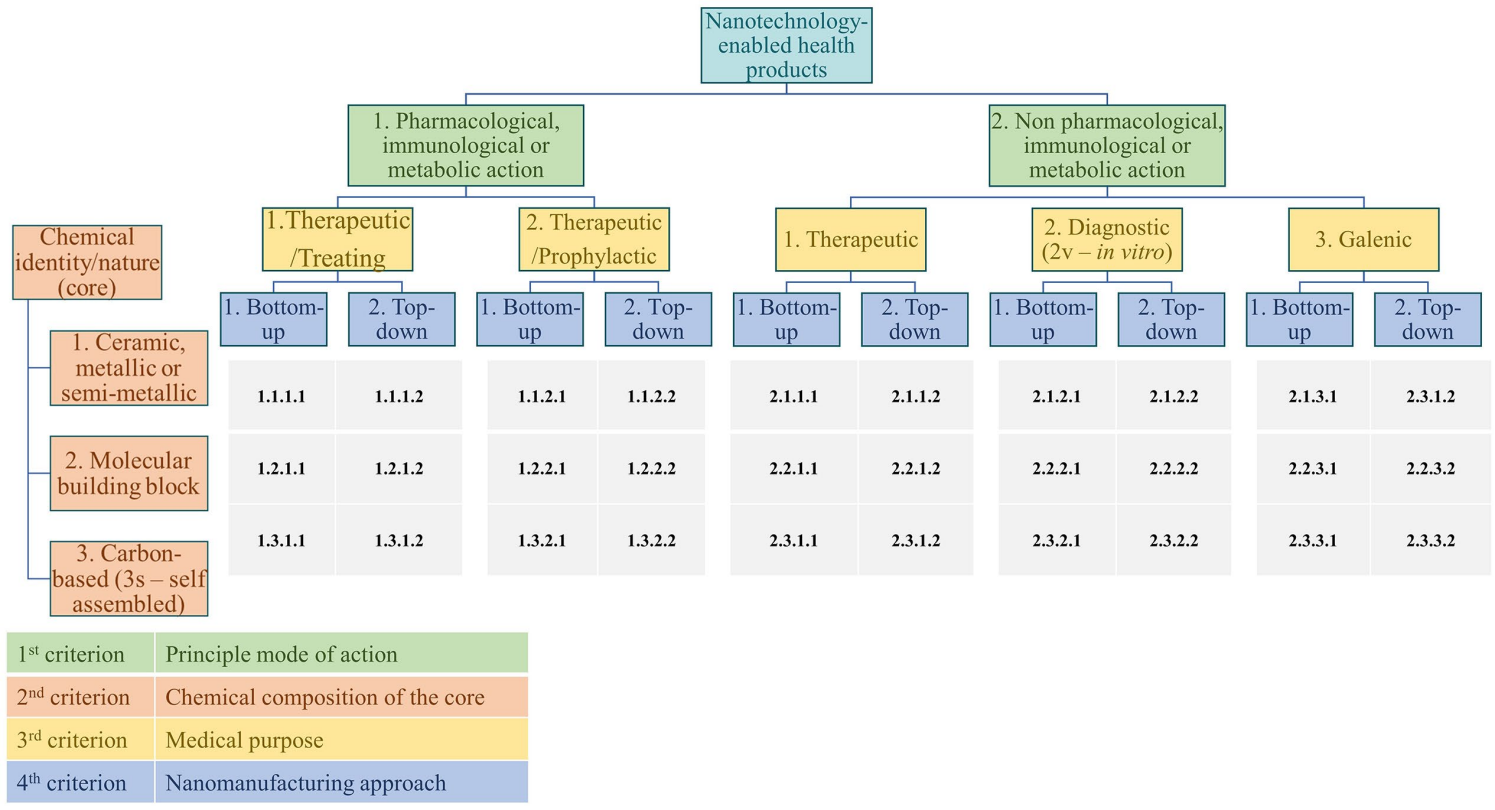
Nanotechnology-enabled health products classification system [taken from Rodríguez-Gómez et al. (22)].

Detected signals on NHP development have been indexed following the classification system described in a previous work of our group (22). This classification system considers both scientific and regulatory criteria and allows the grouping of NHPs into different categories, expected to share similar relevant characteristics when evaluated by regulatory authorities (refer to Figure 1). The system assigns a unique four-digit code, or classification signature, to each category of NHP. This code is based on the NHP’s principal mode of action, chemical composition, intended purpose, and the approach followed in its nanomanufacturing (22).

### 2.4 Data management

Databases have been generated with Excel macro-enabled workbook format (Microsoft Office Professional Plus 2019). Data processing and plotting was carried out by using dynamic tables.

## 3 Results and discussion

### 3.1 Signal detection for Horizon Scanning implementation

The scanners chosen for signal detection included databases that may represent NHPs at various development stages. Data obtained from patent registers typically represent NHPs in the early stages of development. These NHPs might either be newly described or not yet evaluated by a regulatory authority in terms of quality, safety, and performance. Conversely, the clinical studies database represents NHPs that have been assessed by a regulatory authority. These products have already initiated at least initial first-in-human studies. Finally, data obtained from scientific literature databases can cover the entire spectrum: newly developed NHPs or already approved NHPs.

#### 3.1.1 Scopus

Scopus was selected as the database for signal detection among scientific publications. It is a comprehensive database comprising peer-reviewed literature from scientific journals, books, and conference proceedings. Compared to PubMed and Web of Science, Scopus was chosen due to its broader spectrum of journals (23). An extensive literature search was conducted on NHPs with various applications (treatment, diagnosis, prophylaxis). A total of 330 articles were analysed, and 102 articles were selected. The literature search strategy is described in Table 2 and the search results are summarised in Table 3.

**Table 3:** Scan search results.

Scan	String	Scan period	TR	S	NS	Reasons for discarding
Scientific publications – Scopus	(nanomaterial OR nanotechnology) AND (*medicine OR device) AND (treatment OR therapy OR prophylaxis OR diagnos*) AND health* Keywords must appear within title/abstract/keyworks Language: English Limit to document type: article	Scan period 1 01/01/2020–22/06/2021	226	57	169	85 publications were not describing a NHP in particular. 75 publications were not available. 6 publications were outside the selected timeframe. 2 publications were describing applications without medical purposes. 1 publication was not addressing a product made of nanomaterials.
		Scan period 2 23/06/2021–13/01/2022	57	25	32	26 publications were not describing a NHP in particular. 5 publications were not describing applications without medical purposes. 1 publication was not describing a health application within the nano-scale.
		Scan period 3 14/01/2022–15/06/2022	47	20	27	16 publications were not describing a NHP in particular. 6 publications were already covered in the previous scan period. 3 publications were not available. 2 publications were outside the selected timeframe. 1 publication was not describing applications without medical purposes. 1 publication was not addressing a product for human use.
Patents register – European Patent Office (EPO)	EN_TI:(nano*) AND CPC2:(A61)	Scan period 1 01/01/2020–10/08/2021	395	309	86	72 patents were not describing a NHP in particular. 5 patents were duplicated. 5 patents were not under A61 CPC classification. 2 patents were not referring to inventions at the nano-scale. 1 patent was withdrawn. 1 patent was outside the selected timeframe.
		Scan period 2 11/08/2021–13/01/2022	126	99	27	19 patents were not describing a NHP in particular. 6 patents could not be found. 2 patents were duplicated.
		Scan period 3 14/01/2022–15/06/2022	138	116	22	20 patents were not describing a NHP in particular. 2 patents were not under A61 CPC classification.
Clinical studies – Clinicaltrials.gov	Other terms: nano Status: exclude suspended or withdrawn	Scan period 1 not defined-21/07/2021	359	193	166	163 studies were not referring to an investigational product at the nano-scale. 2 studies did not include sufficient data to be considered under the scope. 1 study was not addressing a NHP in particular.
		Scan period 2 22/07/2021–13/01/2022	23	9	14	13 studies were not referring to an investigational product at the nano-scale. 1 study was not referring to an NHP.
		Scan period 3 14/01/2022–15/06/2022	23	8	15	15 studies were not referring to an NHP.

TR: total number of results, S: results selected, NS: results not selected.

#### 3.1.2 Patent register

The European Patent Office (EPO) Database is a crucial resource, providing access to an extensive collection of over 90 million patent documents sourced from more than 90 countries worldwide. This comprehensive database was chosen for signal detection among patents as it illustrates the evolving landscape of global technological advancements, incorporating detailed information on patent applications, patents granted, and their respective status (24). Based on search strategy described in Table 2 a total of 659 patents documents were analysed. From those, 524 were selected for the first stage of signal detection in Horizon Scanning. A summary of the results is listed in Table 3.

#### 3.1.3 Clinical trials

ClinicalTrials.gov is a web-based resource that provides free access to data on publicly and privately supported clinical studies. Sponsored by the U.S. National Library of Medicine, this database offers information about a trial’s purpose, assessed products and participation criteria among other details (25). All clinical studies registered under key prefix nano* were retrieved as described in Table 2. A total of 405 studies were analysed, and 195 were selected for applying the Horizon Scanning methodology. The results are summarised in Table 3.

**Figure 2:**
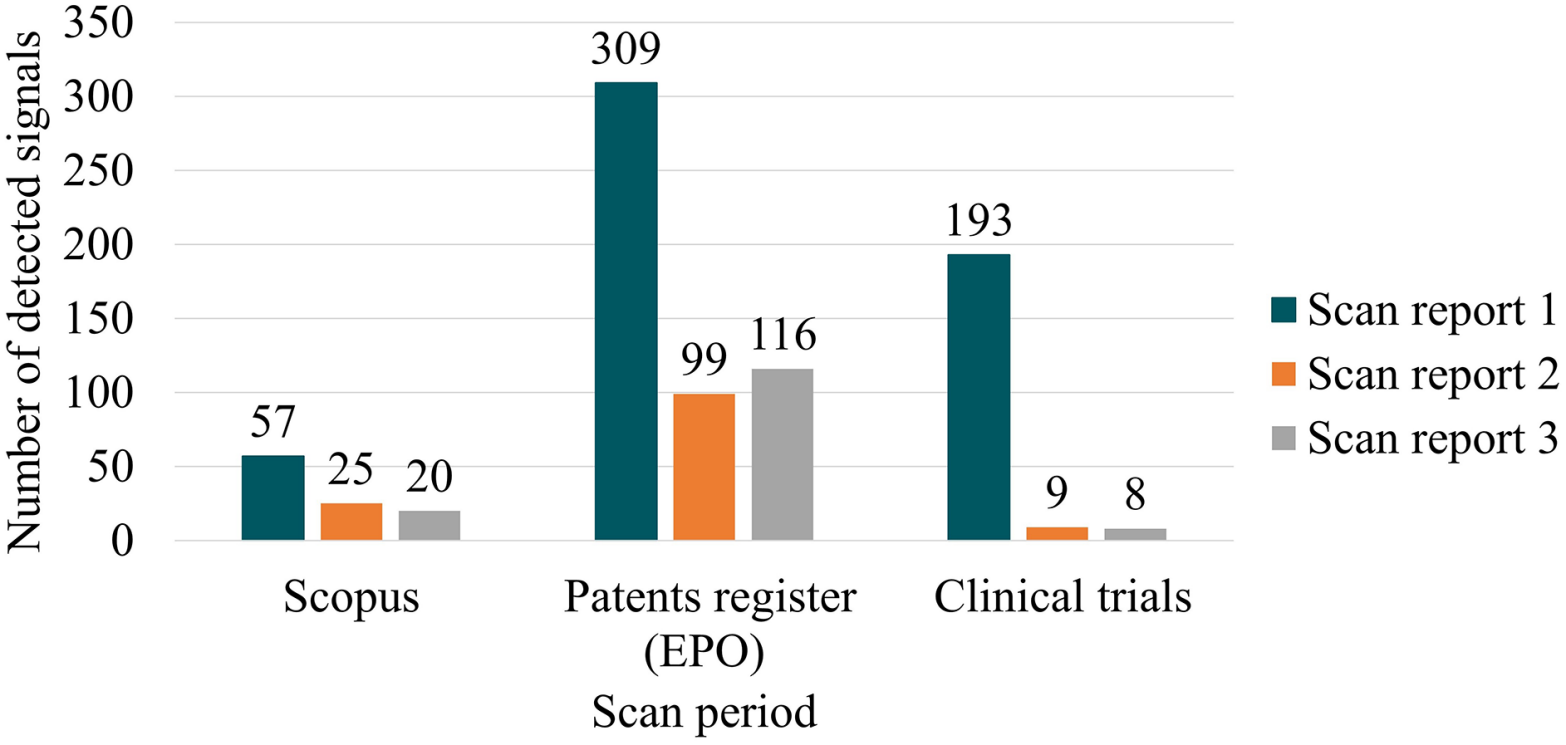
Summary of detected signals for each scan (source of information).

Each detected signal was stored in a database. In this database, every record was linked to its original source of information and classified according to the system defined by Rodríguez-Gómez et al. (22). Figure 2 includes a summary of all the detected signals among the three scans.

### 3.2 Filtration and prioritization of detected signals

#### 3.2.1 Novelty quantification

Novelty was used as a key criterion for filtering and prioritizing the detected signals. A novelty quantification methodology was applied for this purpose. Originally developed for design creativity research, this methodology provides a holistic approach to assess the creativity of various ideas or “design solutions” by contrasting each idea with the entire set of design solutions (20). This structured approach aids objective research in design creativity (21, 26).

**Figure 3:**
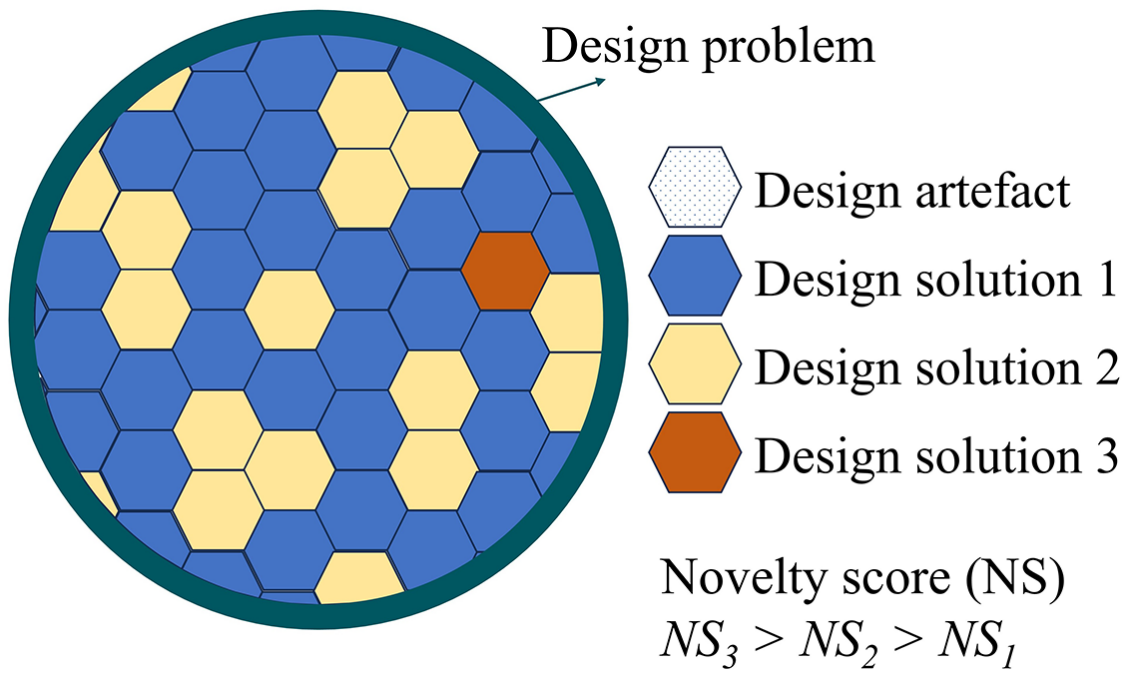
Novelty quantification rationale as per Shah et al. (20).

As proposed by Shah et al. (20), a design problem often leads to the generation of multiple potential solutions, termed as “design artefacts.” Each artefact possesses an “attribute” that outlines its potential to resolve the design problem, referred to as “design solution.” In a situation where various unique solutions are proposed for a design problem, the artefact with the least frequently repeated attribute is considered more novel (Figure 3). For example, given the design problem of developing propulsion systems, if four artefacts are proposed, with three based on sail (design solution 1) and one on jet (design solution 2), the jet-based artefact is deemed more novel due to its uniqueness (20, 27).

Shah et al. (20) methodology has been adapted to quantify the novelty of detected signals. In this context, the various design problems correspond to six categories, which are derived from a combination of two criteria of the applied NHP classification system. Specifically, these criteria are the primary mode of action and medical purpose, resulting in the following design problems:

- Products with pharmacological, immunological, or metabolic action of therapeutic/treating effect.
- Products with pharmacological, immunological, or metabolic action of therapeutic/prophylactic effect.
- Products with non-pharmacological, immunological, or metabolic action of therapeutic effect.
- Products with non-pharmacological, immunological, or metabolic action of not *in vitro* diagnostic effect.
- Products with non-pharmacological, immunological, or metabolic action of *in vitro* diagnostic effect.
- Products with non-pharmacological, immunological, or metabolic action of galenic effect.

Each of these design problems would be the same as the above-mentioned problem of designing a propulsion system and, throughout the design process, NHPs with diverse attributes may be generated to resolve each problem category. For instance, the first category could include NHPs with antifungal and anti-inflammatory actions, while the third might encompass NHPs with structural and moisturizing actions. Each attribute corresponds to a distinct design solution. Supplementary Table S1 provides a comprehensive list of all design solutions (identified by a design solution code, DSC) defined for all NHPs in the detected signals.

**Figure 4:**
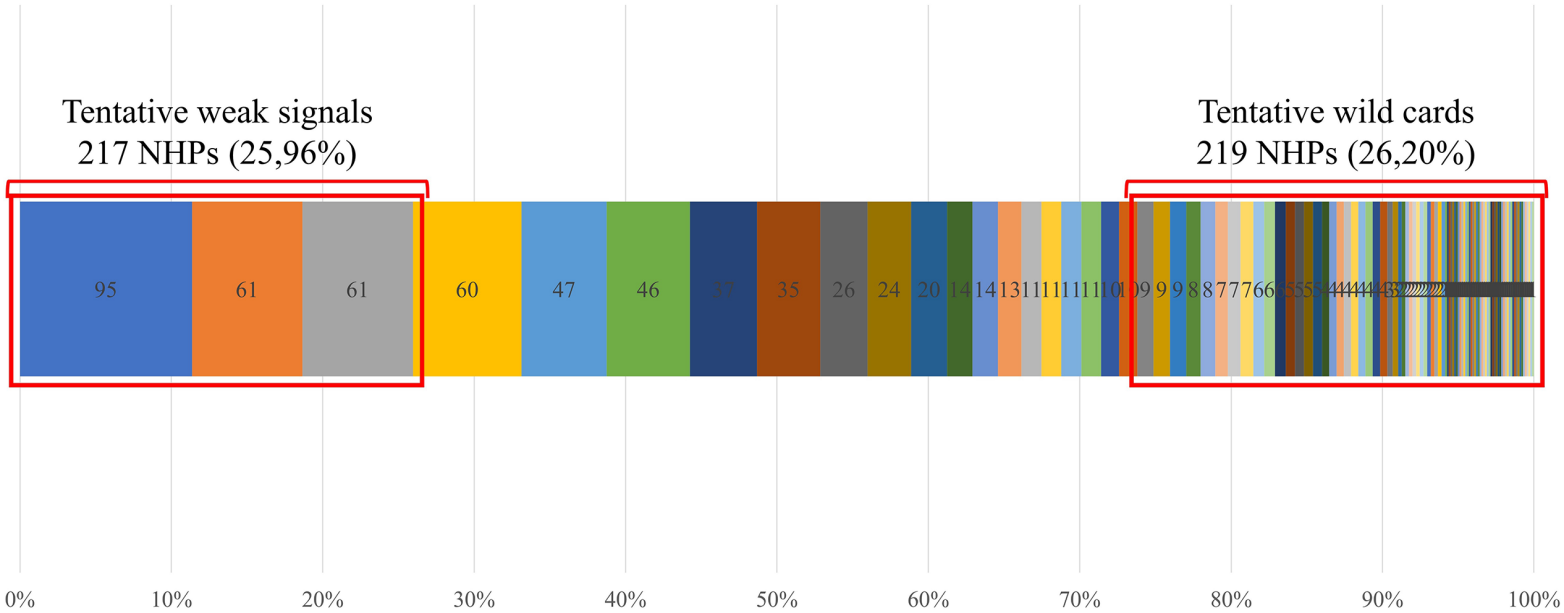
Cumulative percentage of the frequency of design solutions.

For each detected signal, the corresponding NHP and DSC are determined, and novelty is quantified according to Eq. 1. Signals with a novelty score of 10 or closer are deemed more novel. Additionally, the cumulative percentage of the frequency of design solutions can be displayed in descending order of frequency per design solution. This approach, as established by Sluis-Thiescheffer et al. (27), allows setting the third quartile (Q3) as the threshold to identify the most disruptive and novel elements (wild cards), while the first quartile (Q1) can denote trends in design solutions. Figure 4 provides an overview of this representation encompassing the complete spectrum of identified signals sourced from scientific literature, patent registries, and clinical study databases.

Novelty score calculation:


(1) $$S_{1}=\frac{T-C_{1}}{T}\cdot 10;\forall T,C\in N$$

S_1_ is the “novelty score” for design solution 1. C_1_ is the frequency for design solution 1. T is the total number of design solutions.

Novelty analysis of the design solutions of detected signals. Design solutions are in descending order of frequency (i.e., in ascending order of novelty). The red squares show the 0.25 and 0.75 percentiles, Q1 and Q3, respectively. All design solutions with a frequency higher or equal to Q1 are considered tentative trends, all design solutions with a frequency lower or equal to Q3 are considered tentative wild cards.

#### 3.2.2 Potential trends in NHPs

**Figure 5:**
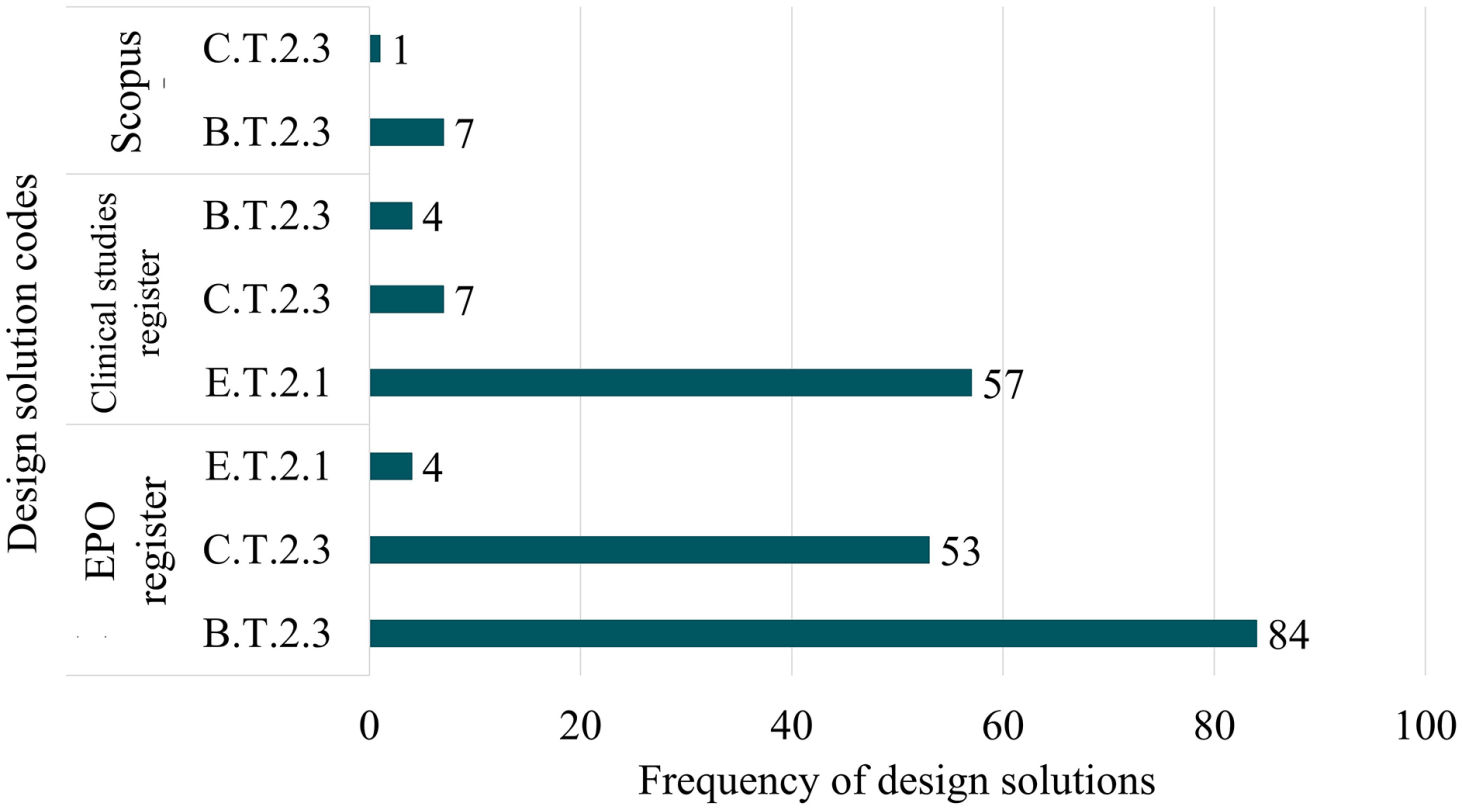
Tentative weak signal distribution among sources for signal detection.

As initially outlined, the identification of trends among all detected signals is determined by the frequency distribution of each DSC. NHPs whose DSCs fall within the top quartile of the total distribution are classified as trends, or weak signals. Figure 4 shows that frequencies exceeding the Q1 correspond to three distinct DSCs, representing 217 of the 836 detected NHPs. Table 4 compiles the DSCs classified as trends, while Figure 5 presents the distribution of NHPs linked to these trends across various information sources.

**Table 4:** Tentative weak signals (≤ Q1).

C	DSC	NS	DSC descriptor	DSC definition	Examples*
95	B.T.2.3	8,86	Molecular building block nanocarrier	Drug carrier made of polymers (except those made of protein and nucleic acid that constitute an independent design solution).	Chitosan, acrylate or polysaccharidic nanoparticles.
61	E.T.2.1	9,27	Dental filling/replacement material	Nanomaterial used for the treatment of dental cavities or tooth defects.	Ceramic resins, glass ionomer, nanometallic varnish or hydroxyapatite nanoparticles.
61	C.T.2.3	9,27	Lipid nanocarrier	Drug carrier made of lipids, in a broad sense.	Lipid nanoparticles, solid lipid nanoparticles or lipid nanocapsules.

C: frequency of each design solution, DSC: design solution code, NS: novelty score. *Non-exhaustive list with more representative examples for each weak signal.

Based on the results, a clear trend towards the development of drug delivery systems (DDS) is evident. The most prevalent systems are based on polymer (DSC: B.T.2.3) and lipid (DSC: C.T.2.3) chemistry (95 and 61 selected signals respectively). Moreover, a growing trend in the development of nanomaterials for dental applications (DSC: E.T.2.1), such as surface filling or tooth replacement, is also notable (61 selected signals).

**Figure 6:**
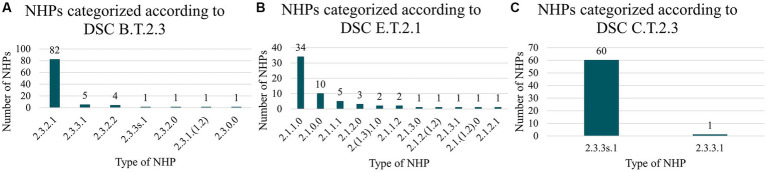
NHP classification code of signals identified as trends (weak signals). **(A)** Represents NHPs for trends identified as *molecular building block nanocarrier* (B.T.2.3). **(B)** Represents NHPs for trends identified as *dental filling/replacement material* (E.T.2.1). **(C)** Represents NHPs for trends identified as *lipid nanocarrier* (C.T.2.3).

Figure 6 summarises the types of NHPs associated with the DSCs B.T.2.3, C.T.2.3, and E.T.2.1. As expected, all identified NHPs linked to DDS, i.e., B.T.2.3 and C.T.2.3, possess a classification signature featuring “2.3.n.n”, indicating a non-pharmacological, immunological, or metabolic action (“2.n.n.n”) and a galenic function (“n.3.n.n”). For NHPs with DSC B.T.2.3, those with a classification signature “2.3.2.1” predominate. This indicates a molecular building block composition (“n.n.2.n”) and a bottom-up nanofabrication process (“n.n.n.1”). For NHPs with DSC C.T.2.3, carbon-based NHPs, particularly those with self-assembling structures in physiological medium (“n.n.3s.n”), are dominant. All these NHPs are produced using a bottom-up nanofabrication approach (“n.n.n.1”).

In the case of NHPs with DSC E.T.2.1, dental filling/replacement material, all detected signals correspond to NHPs with a primary action that is not based on pharmacological, immunological, or metabolic means and whose medical indication is therapeutic (signature featuring “2.1.n.n”). The chemical composition varies, with metallic (“n.1.n.n”) composition being predominant, followed by polymeric (“n.2.n.n”), carbon-based (“n.3.n.n”), or occasionally, undefined compositions (“n.0.n.n”). The nanofabrication approach is often undefined, but some NHPs are produced using bottom-up (“n.n.n.1”), top-down techniques (“n.n.n.2”), or combinations thereof.

Overall, the three DSCs identified as trends correspond to NHPs with relatively consistent classification signatures.

The increasing interest within the medical community towards DDS reflects their potential to significantly transform therapeutic interventions (28, 29), particularly in the principal therapeutic areas of nanotechnology development, which are predominantly cancer and then infectious diseases (1, 2). These complex systems serve to improve solubility, encapsulation efficacy and therefore the pharmacokinetic properties of medicinal products. In addition, DDS can be designed for targeting specific cells or tissues, thereby enhancing efficacy while reducing systemic side effects (30). DDS have the ability to control the time and location of drug release, assuring a consistent therapeutic effect and overcoming the limitations of traditional dosage forms. Moreover, DDS can be designed to overcome biological barriers, enabling the delivery of a wider range of therapeutics, including genes and biologics. Eventually, it is estimated that 90% of new medicinal products developed by the pharmaceutical industry are discarded despite their effectivity due to solubility issues (31). In response to the decreasing number of new pharmaceuticals, there is a growing trend towards exploring alternative drug delivery methods to increase the safety and efficacy of existing pharmaceuticals (31, 32). In addition, the global pharmaceutical drug delivery market, indicative of the potential and growth of DDS, is projected to reach a staggering USD 2206.5 billion by 2026, exhibiting a CAGR of 5.9% (33). In this context, lipid and polymer platforms have been identified as trends in NHP development based on Horizon Scanning methodology (Table 4).

The integration of nanomaterials into dentistry represents a significant advancement in enhancing oral health care. These nano-based structures, with their unique physicochemical properties, have led to the development of innovative dental materials and techniques. Nanocomposites and nanoceramics, for example, offer excellent aesthetic and mechanical properties, thereby improving the durability and appearance of restorations. Furthermore, the application of nanotechnology in dentistry goes beyond restorative materials, with nanoparticle-based antimicrobials offering a promising strategy in combating oral biofilms (34).

Importantly, many nanomaterial applications in dentistry are based on structural action, suggesting possible classification within the regulatory category of medical devices in the European Union (35). This hypothesis is backed by the majority of detected signals related to these types of products coming from clinical trial registries (Figure 5). This is also in agreement with the fact that medical devices undergo a shorter regulatory preclinical development compared to pharmaceuticals, thus enabling earlier evaluation in human studies.

#### 3.2.3 Potential wild cards in NHPs

**Figure 7:**
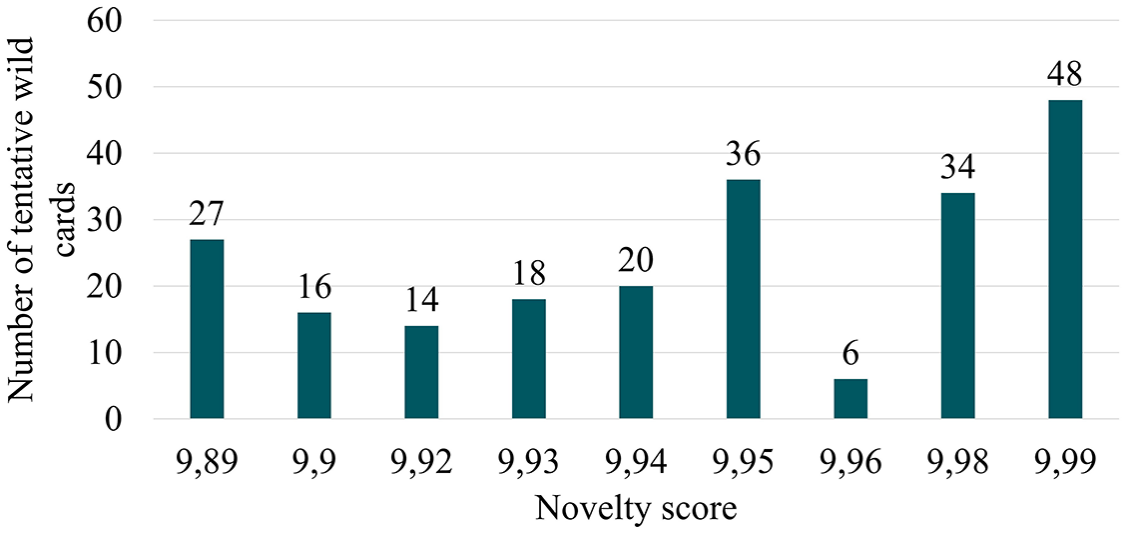
Novelty score distribution of tentative wild cards.

Among the total detected signals, the most innovative NHPs correspond to those with the highest novelty scores. These are the NHPs with design solutions falling within the third quartile as shown in Figure 4. However, when analysing the 219 results obtained, prioritizing some NHPs over others in terms of novelty proved challenging due to the similar and near-maximum (between 9.89 and 9.99) novelty scores (Figure 7). Hence, a novelty-enrichment method has been proposed to adjust the novelty scores based on various criteria. Table 5 presents the criteria employed for novelty-correction and the rationale for each.

**Table 5:** Correction factors for novelty score of tentative wild cards.

Question	Answers		Correction factor	Justification
Does the tentative wild card include a DSCs with “Y” or “Z”?*	Z1	Yes	0	Unspecific DSCs shall be removed as no conclusions can be made from them.
	Z2	No	1	
How many DSCs are used for defining the tentative wild card?	N1	1	1	Multi-composition/multifunctionality of NHPs shall be enriched in terms of novelty score.
	N2	2	3/2	
	N3	> 2	2	
How many DSCs are the same as one of a weak signal?	S1	0	2	NHPs sharing DSCs with weak signals (trends) shall be penalised in terms of novelty score. NHPs not sharing DSCs with weak signals shall be enriched.
	S2	1	2/3	
	S2	> 1	1/2	
Does the tentative wild card include a NHP defined among the weak signals?	C1	Yes	1	Tentative wild cards not sharing the same NHP shall be enriched in terms of novelty score.
	C2	No	2	

*Refer to Supplementary Table S1. “Y” and “Z” design solution codes correspond to detected signals where the attribute is various, i.e., not concrete, or it is not sufficiently defined, respectively. DSC: design solution code. NHP: nanotechnology-enabled health product.

**Figure 8:**
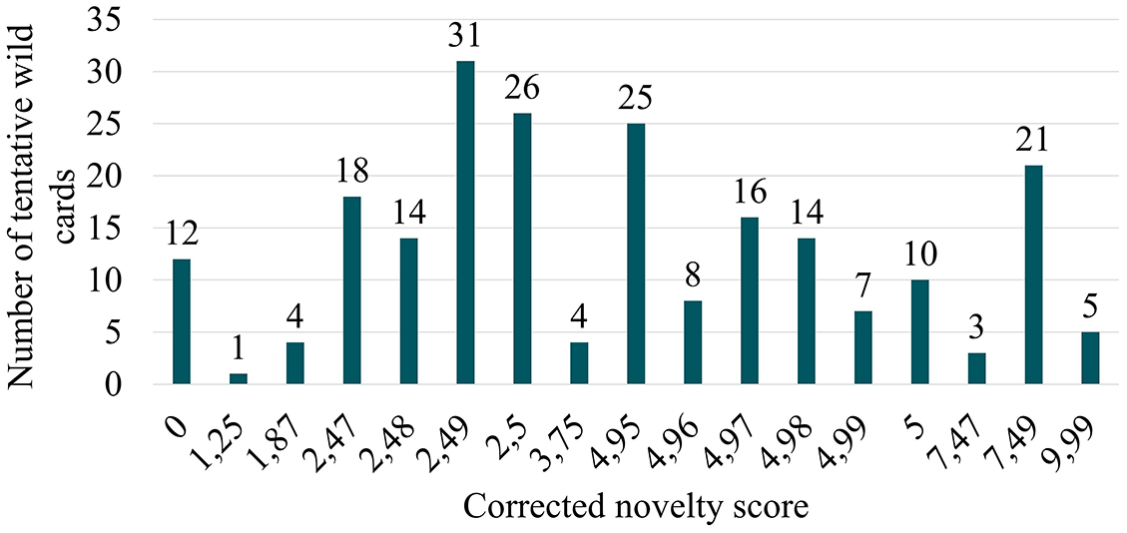
Corrected novelty score distribution of tentative wild cards.

After implementing this adjustment, the distribution of corrected novelty scores becomes segregated (Figure 8). Consequently, five NHPs with the highest novelty scores are identified. These are NHPs whose combinations of design solutions appeared only once in the total of 836 detected signals (Table 6). It can be inferred that the NHPs identified as the most innovative (wild cards) correspond to systems integrating nanomaterials with diverse chemical compositions, capable of possessing various therapeutic, diagnostic, or galenic effects in a single entity.

**Table 6:** Wild cards (≥ Q3).

DSC	DSC descriptor	DSC definition	NHP	NS*
F.T.2.1*and*A.T.2.2*and*P.T.2.3	Photothermal therapyNaked eye detectionRadiosensitive nanocarrier	Generation of heat upon light stimulationSubstances that are injected so that they distribute and accumulate in certain regions allowing it identification (e.g., lymph tracer). Near-Infrared Imaging (NIR) imaging is an example of this type of detection.Smart nanoparticle that activates when exposed to a radiation field.	2.(1.2.3).1.1	9,99
K.T.1.1*and*F.T.2.1*and*E.T.2.2					Antitumoral activityPhotothermal therapyMultimodal imaging	Cytotoxic effect broadly applicable for the treatment of cancer (not necessarily limited to reactive oxygen species-ROS-generation).Generation of heat upon light stimulation.Substances used for simultaneous production of signals for more than one imaging technique.	1.1.(2.3).1	9,99
B.T.1.1*and*G.T.2.1*and*L.T.2.3	Antibacterial actionRadiation sensitizerMagnetic-field targeted nanocarrier	Antibacterial action in a broad sense based on pharmacological means.Generation of heat upon radiation for radio-therapy enhancement.Magnetic nanoparticle that can be guided or activated (e.g., warmed) based of magnetic field exposure.							1.1.(1.2).1	9,99
B.T.1.1*and*H.T.1.1*and*G.T.2.1					Antibacterial actionPhotodynamic therapyRadiation sensitizer	Antibacterial action in a broad sense based on pharmacological meansROS generation upon photo-stimulationGeneration of heat upon radiation for radio-therapy enhancement					(1.2).1.1.1	9,99
R.T.2.1*and*B.T.2.2*and*L.T.2.3	ThermotherapyMRI contrast agentMagnetic-field targeted nanocarrier	Substances that are heated up and used for thermotherapy.Substances for improving visibility in medical imaging based on magnetic resonance imaging (MRI).Magnetic nanoparticle that can be guided or activated (e.g., warmed) based of magnetic field exposure.											2.(1.2.3).1.1	9,99

DSC: design solution code, NHP: nanotechnology-enabled health product, NS*: novelty score corrected.

The emergence of multicomponent and multifunctional nanomaterials in healthcare signifies a transformative leap in medical therapeutics and diagnosis (36). These complex structures offer enhanced functionality by facilitating the concurrent delivery of diverse therapeutics and diagnostic agents. This multifunctionality not only amplifies therapeutic efficacy, but also enables real-time monitoring of treatment efficacy, representing the essence of theranostics (37). Furthermore, these nanomaterials can be engineered to treat pathologies with multiple mechanisms of action simultaneously (38, 39). This capacity is largely attributed to the unique properties that materials exhibit at the nanometric scale (40), providing a level of therapeutic control unparalleled in conventional treatment modalities. Consequently, the exploration of these sophisticated nanomaterials heralds a promising future in personalised medicine, paving the way for more efficient and customised healthcare solutions (41).

### 3.3 Assessment

In the final phase of the Horizon Scanning process, signals detected date from June 2022. There exists roughly a one-year gap between the last update of the regulatory database (August 31st, 2023) and the detection of these signals. However, this gap is not expected to considerably affect the conclusions drawn. As previously stated, the rate of innovation in NHPs outpaces the progress of their regulations. Thus, despite the one-year difference, there may be areas still insufficiently regulated, as highlighted in this study.

#### 3.3.1 Regulatory considerations for dental and galenic NHPs

In broad terms, the European legal basis of NHPs intended for dental use, classified under the DSC E.T.2.1, is the *Regulation (EU) 2017/745 of the European Parliament and of the Council of 5 April 2017 on medical devices* which refers to products with a medical purpose exerting a physical/mechanical primary mode of action. In the regulatory database, only 2 documents were found to directly address NHPs under E.T.2.1: “ISO/TS 23367-1:2022 – Nanotechnologies — Performance characteristics of nanosensors for chemical and biomolecule detection – Part 1: Detection performance” and the European Commission’s “Opinion on Titanium Dioxide (nano form) as UV-Filter in sprays” (refer to the complete Regulatory database attached in Supplementary Table S2). However, it is important to note that none of these documents refer to dental application products.

Contrarily, in Europe DDS are governed by *Directive 2001/83/EC of the European Parliament and of the Council of 6 November 2001 on the Community code relating to medicinal products for human use*. Table 7 provides a thorough list of all identified regulatory documents specific to NHPs with a galenic action. Out of the 9 references, 6 are dedicated to nanoliposomes (NHP signature 2.3.3 s.1) and 1 document is addressing polymer-based DDS (NHP signature 2.3.2.1).

**Table 7:** Regulatory documents specifically applicable to NHPs with a galenic function.

Source	Status	Name	Type of NHP
ASTM	Under development	ASTM WK75607 – New Guide for Characterization of Encapsulation, Extraction, and Analysis of RNA in Lipid Nanoparticle Formulations for Drug Delivery	2.3.3 s.1
ASTM	Under development	ASTM WK67980 – New Test Method for Quantifying Poly (ethylene glycol) Coating on the Surface of Gold Nanostructured Materials Using High Performance Liquid Chromatography with Evaporative Light Scattering Detection (HPLC/ELSD)	2.3.1.0
ASTM	Under development	ASTM WK68060 – New Test Method for Analysis of Liposomal Drug Formulations using Multidetector Asymmetrical-Flow Field-Flow Fractionation (AF4)	2.3.3 s.1
European Commission	Current version	Analytical ultracentrifugation for measuring drug distribution of doxorubicin loaded liposomes in human serum	2.3.3 s.1
ISO	Under development	ISO/CD TS 4958 – Nanotechnologies — Liposomes terminology	2.3.3 s.1
FDA	Final	Liposome Drug Products: Chemistry, Manufacturing, and Controls; Human Pharmacokinetics and Bioavailability; and Labeling Documentation	2.3.3 s.1
EMA	Current version	Reflection paper on surface coatings: general issues for consideration regarding parenteral administration of coated nanomedicine products	2.3.0.1
EMA	Current version	Joint MHLW/EMA reflection paper on the development of block copolymer micelle medicinal products	2.3.2.1
EMA	Current version	Reflection paper on the data requirements for intravenous liposomal products developed with reference to an innovator liposomal product	2.3.3 s.1

A closer look at the Nanotechnology Product Database (NPD), recognised as a reliable resource for institutions and policymakers establishing national policies and strategic plans (42), reveals a specific category for dental applications of NHPs. This category includes a total of 190 approved products worldwide (as of 11 December 2023). The NPD website has also a dedicated section for pharmaceutical products, which includes a total of 432 approved products worldwide (as of 11 December 2023). In this section, products utilising polymeric nanoparticles and nanoliposomes are the most common among the approved products (59 and 15, respectively).

This information suggests that despite the relatively low number of specific regulatory documents for NHPs for dental applications or galenic action (based on polymers and lipids), these types of NHPs have most frequently achieved regulatory approval. One possibility for this is that development and evaluation of these NHPs may have been based on other more general, less specific regulatory guidelines to achieve regulatory approval.

On the one hand, nano-filled composite dental materials demonstrate superior physical properties, such as compressive strength, tensile strength, toughness, flexural strength, and abrasion resistance, compared to their micro-particle filled counterparts. Historically, nanocomposite materials have been utilised in dentistry, and this remains a growing area of research and innovation (34). Given its established nature, regulatory hurdles are not anticipated in this specific area.

On the other hand, although polymer and lipid-based nanoparticles appear to be achieving more regulatory approval success due to extensive experience and knowledge within the scientific community, along with their generally optimal controlled-release and stability profile, there are still substantial challenges for DDSs (43). These challenges mainly include the need for safety and biological behaviour assessment prior to approval. In biological environments, the constituents of the medium are typically adsorbed onto the nanoparticle surface due to their small size, creating a hybrid system where the nanoparticle becomes enveloped with organic particles, predominantly proteins, forming a corona (44, 45). This phenomenon alters the expected pharmacokinetic compartment for small drug molecules, which is not equivalent for NHPs (46). The connection between the physicochemical properties of NHPs and their pharmacokinetic behaviour and safety profile requires a more thorough understanding, and is considered one of the main challenges in meeting regulatory expectations (47–49). In fact, in light of these complexities, the EMA has underscored the importance of establishing guidelines for the evaluation of pharmacodynamics and pharmacokinetics of NHPs, as a fundamental part of its regulatory science strategy towards 2025 (3).

#### 3.3.2 Regulatory considerations for multifunctional NHPs

The innovation of nanotechnology is introducing a new era of multifunctional and multicomposite NHPs, each with unique properties and potential applications (50). These innovative products are often developed by adopting one of two primary strategies, commonly referred to as the “one-for-all” and “all-in-one” approaches. The “one-for-all” approach involves the development of a single building block with inherent multifunctional properties (51, 52), whereas the “all-in-one” approach combines different agents into a single nanoparticle with distinct sections, each serving a different function (29, 39, 53).

NHPs produced using the “all-in-one” approach are often designed with stealth-like features to evade the immune system and prevent opsonization, protective layers to prevent degradation, targeting moieties for improved specificity, membrane-permeation moieties for enhanced cell uptake, as well as imaging agents for delivery and dosing control, and monitoring. However, each additional functionality increases complexity and cost, particularly due to the heterogeneous nature of the formulations (38). Notwithstanding, “all-in-one” NHPs offer the opportunity to utilise components that have already been approved or have other healthcare applications, potentially mitigating challenges for regulatory approval. In contrast, the “one-for-all” approach often requires the exploration of novel materials with inherent multifunctionality, which can complicate regulatory approval due to their novelty (39).

Despite their complexities, multifunctional NHPs offer considerable advantages such as the potential to combine different imaging techniques that provide complementary information, or to deliver substances with synergistic mechanisms of action that can enhance treatment efficacy. However, no specific regulatory guidelines have been identified that address specifically multifunctional NHPs. These disruptive elements, indeed, mandate strategic reinforcement to existing regulations. The goal shall be to facilitate the development of these innovative health products, ensure their comprehensive evaluation, and realise their full benefits, all the while ensuring patient safety. As such, filling up the current regulatory landscape becomes essential to appropriately address these uniquely complex and promising products.

## 4 Conclusion

To conclude, a notable disparity exists between the innovative medical products being developed and those that achieve regulatory approval, especially in the case of NHPs. This is primarily due to the lack of adequate regulatory guidelines that can effectively evaluate the quality, safety, and efficacy of these products. The application of Horizon Scanning methodology, a standard practice among legislators and health authorities, can be key for anticipating future regulatory needs. Using Horizon Scanning methodology, this study identifies DDSs and dental applications as leading trends in NHP development. In turn, multifunctional and multicomposite products are identified as the most novel.

While the regulatory landscape is becoming more accommodating for emerging trends, substantial challenges still exist for the most innovative technologies. As regulatory science works to keep up with technological innovation, the continued focus on developing robust and adaptable regulatory guidelines for NHPs is of utmost importance. This will facilitate the safe and effective transition of these groundbreaking products from the research stage to clinical application, ultimately serving the best interests of patients and propelling the progress of nanomedicine.

## Data availability statement

The raw data supporting the conclusions of this article will be made available by the authors, without undue reservation.

## Author contributions

FDRG: Conceptualization, Data curation, Formal analysis, Investigation, Methodology, Project administration, Visualization, Writing – original draft. DM: Conceptualization, Funding acquisition, Project administration, Supervision, Validation, Writing – review & editing. OP: Conceptualization, Funding acquisition, Project administration, Supervision, Validation, Writing – review & editing. PRG: Conceptualization, Funding acquisition, Supervision, Validation, Writing – review & editing.

## Footnotes

^1^“Compound Annual Growth Rate (CAGR)” average yearly growth rate over a specified period of time, providing a smoothed annual growth perspective.

^2^Nanotechnology-enabled health product’ is an overarching term for nanomedicines (health products including nanomaterials that are regulated as medicinal products) and nanomedical devices (health products including nanomaterials that are regulated as medical devices).
