# Supplementary material for: Implementing Horizon Scanning as a tool for the strategic development of regulatory guidelines for nanotechnology-enabled health products
Source: Front Med (Lausanne). 2024 Jan 11;10:1308047. doi: 10.3389/fmed.2023.1308047 (PMC10829765; doi:10.3389/fmed.2023.1308047)
Supplement: Supplementary file 1 [file Table_1.pdf]

Implementing Horizon Scanning as a Tool for the Strategic Development of Regulatory Guidelines for Nanotechnology-Enabled Health Products

Francisco D. Rodríguez-Gómez[1,2*], Dominique Monferrer[1], Oriol Penon[1], Pilar Rivera-Gil[2]

[1]Asphalion SL, Barcelona, Spain

[2]Integrative Biomedical Materials and Nanomedicine Lab, Department of Medicine and Life Sciences, Universitat Pompeu Fabra Barcelona Biomedicine Research Park (PRBB) Doctor Aiguader, 88, Barcelona, Spain

*** Correspondence:**
Corresponding Author
franciscodiego.rodriguez01@estudiant.upf.edu

Keywords: Regulatory Science, Horizon Scanning, Nanomedicines, Nanotechnology-Enabled Health Products, Regulatory Guidelines.

SUPPLEMENTARY MATERIAL

Supplementary Table 1: Design solutions identified for selected signals.

Code	Descriptor	Definition	Code	Descriptor	Definition	Code	Descriptor	Definition
Products with pharmacological, immunological, or metabolic action of therapeutic/treating effect			I.T.2.1	Moisturizer / Emollient	Moisturizing or emollient substances at the nanoscale	B.T.2.2v	Nanomaterials for 3D cell culture	Nanomaterials generating 3D-structure for mimetic in vivo conditions in cell cultures
A.T.1.1	Gene editing or modification of the gene expression	Gene editing for the modification of gene expression (e.g., CRISPR)	J.T.2.1	Bone grafting material	Nanomaterial used for filling bone defects, mimeting extracellular bone matrix	C.T.2.2v	Nano adsorbent	Substances attached to a platform that specifically adsorbs other substances allowing its detection (e.g. antibodies attached in a surface in lateral flow assays)
B.T.1.1	Antibacterial action	Antibacterial action in a broad sense based on pharmacological means	K.T.2.1	Sunscreen	Physical or chemical sunscreen at the nanoscale	D.T.2.2v	Photothermal assay	A probe with photothermal properties when attached to a given substrate. Positive signal is translated in an increased temperature that can be measure with a Forward-looking infrared (FLIR) camera.
C.T.1.1	Anti-inflammatory action	Anti-inflammatory action in a broad sense based on immunological means	L.T.2.1	Acoustic therapy	Generation of physical stress (movement/heat) upon sound stimulation	Y.T.2.2v	Various	
D.T.1.1	Antiviral action	Antiviral action in a broad sense based on pharmacological means	M.T.2.1	Optical droplet vaporization	Generation of air droplets upon light induction	Z.T.2.2v	Not sufficiently defined	

Code	Descriptor	Definition	Code	Descriptor	Definition	Code	Descriptor	Definition
E.T.1.1	Metabolite-based supplementation	Enhancement of physiological functions based on the supplementation of metabolites or substrates for involved biochemical processes	N.T.2.1	Nanoparticle-assisted laser tissue soldering	Solder nanomaterial that upon light induction heats and allowing soldering of tissues	Products with non-pharmacological, immunological, or metabolic action of galenic effect		
F.T.1.1	Immunological modulation	Modulation of the immune system based on the use of (modified) immune-mediators such as Igmo or T-CARs cells	Ñ.T.2.1	Structural action	Nanomaterials used solely as a construction material of medical devices	A.T.2.3	Protein-based nanocarrier	Drug carrier made of poly/oligopeptides (nanocapsules, drug binded to a nanoprotein, etc)
G.T.1.1	ROS-mediated cytotoxicity	Generation of ROS for inducing cell lysis (typically related to a antitumoral activity)	O.T.2.1	Cryotherapy	Substances that are cooled down and used for cryotherapy	B.T.2.3	Molecular building block nanocarrier	Drug carrier made of polymers (except protein or nucleic acids which are specifically addressed by DSC A.T.2.3 and Ñ.T.2.3, respectively).
H.T.1.1	Photodynamic therapy	ROS generation upon photo-stimulation	P.T.2.1	Radioisotope chelator	Substance used for chelating radioisotopes at an specific location	C.T.2.3	Lipid nanocarrier	Drug carrier made of lipid, in a broad sense
I.T.1.1	Tissue regeneration enhancer	Technology containing bioactive signals that induce healing (e.g. by recruitment of immune cells)	Q.T.2.1	Radioprotector	Substances used for radiation protection	D.T.2.3	Nanoemulsion-based nanocarrier	Carrier consisting of nanodroplets (including both, W/O or O/W nanoemulsions)
J.T.1.1	Cell protection	Attenuation of toxicity	R.T.2.1	Thermotherapy	Substances that are heated up and used for thermotherapy	E.T.2.3	Lipid bilayer nanocarrier	Carrier consisting of a lipid bilayer nanovesicle

Code	Descriptor	Definition	Code	Descriptor	Definition	Code	Descriptor	Definition
K.T.1.1	Antitumoral activity	Cytotoxic effect broadly applicable for the treatment of cancer (not necessarily limited to ROS generation)	S.T.2.1	*In vivo* circulating cell adhesion	Substances that bind targeted cell types *in vivo* and induce their accumulation or removal	F.T.2.3	Prevention from fogging or smearing	Lents coating designed for preventing fogging or smearing
L.T.1.1	Miniaturized existing medicine	Free medicines at the nanoscale that are part of a dispersed system wherein they are included in a dispensing carrier agent (e.g. dispersion, aerosol, etc.)	T.T.2.1	Upconverting nanoparticles	Particles that exhibit photon up conversion. This is a process in which the sequential absorption of two or more photons leads to the emission of light at shorter wavelength than the excitation wavelength.	G.T.2.3	Carbon-based nanocarrier	Drug carrier made of organic molecules or molecules with a carbon skeleton
M.T.1.1	Hemostatic agent	Substances that induce blood clotting	U.T.2.1	Renal clearance	Particles intended to undergo renal clearance	H.T.2.3	pH-responsive nanocarrier	Smart nanoparticle that activates when a certain pH is achieved - physical trigger
N.T.1.1	Lipid in vivo transport	Physiological lipid transport mimetic	W.T.2.1	Oscillation	Particle induced oscillation for mechanical ablation of cells	I.T.2.3	Metal nanocarrier	Drug carrier made of metal
Y.T.1.1	Various		Y.T.2.1	Various		J.T.2.3	Oxygen-responsive nanocarrier	Smart nanoparticle that activated when a certain temperature is achieved
Z.T.1.1	Not sufficiently defined		Z.T.2.1	Not sufficiently defined		K.T.2.3	Temperature-responsive nanocarrier	Smart nanoparticle that activates when a certain temperature is achieved
Products with pharmacological, immunological, or metabolic action of therapeutic/prophylactic effect			Products with non-pharmacological, immunological, or metabolic action of diagnostic effect			L.T.2.3	Magnetic-field targeted nanocarrier	Magnetic nanoparticle that can be guided or activated (e.g., warmed) based of

Code	Descriptor	Definition	Code	Descriptor	Definition	Code	Descriptor	Definition
								magnetic field exposure
A.T.1.2	Vaccine adjuvant	Material which is incorporated with a vaccine for the purpose of enhancing the immune response of the vaccine	A.T.2.2	Naked eye detection	Substances that are injected so that they distribute and accumulate in certain regions allowing it identification (e.g. lymph tracer). NIR imaging is an example of this type of detection	M.T.2.3	Antioxidant nanocarrier	Nanoparticle that acts as a ROS scavenger
Y.T.1.2	Various		B.T.2.2	MRI contrast agent	Substances for improving visibility in medical imaging based on MRI	N.T.2.3	Electric-field responsive nanocarrier	Nanoparticle that changes its configuration upon electrical stimulation
Z.T.1.2	Not sufficiently defined		C.T.2.2	Photoluminescence contrast agent	Substances with inner luminescent properties	Ñ.T.2.3	Nucleic acid nanocarrier	Drug carrier made of nucleic acids, in a broad sense
Products with non-pharmacological, immunological, or metabolic action of therapeutic effect			D.T.2.2	PET / SPECT contrast agents	Substances for improving visibility in medical imaging based on PET/SPECT	O.T.2.3	Ultrasound-responsive nanocarrier	Smart nanoparticle compromising nanobubbles loading with medicinal products that release upon ultrasound induction
A.T.2.1	Wound dressing	Film/coverage to physically protect injuries from external agents. Typically, these protective films ensure optimal conditions for tissue healing are maintained.	E.T.2.2	Multimodal imaging		P.T.2.3	Radiosensitive nanocarrier	Smart nanoparticle that activates when exposed to a radiation field
B.T.2.1	*In vivo* monitoring	Invasive or body contacting technologies which are aimed	F.T.2.2	Photoacoustic imaging	Substances for improve visibility in medical imaging based on echography	Q.T.2.3	Biological barrier disruptor	Smart carrier designed to disrupt/alter biological barrier

Code	Descriptor	Definition	Code	Descriptor	Definition	Code	Descriptor	Definition
		at monitoring different parameters. They are not considered contact agents as they would have a diagnostic action			(ultrasound contrast agents)			upon stimulation making it permeable to cargo substances
C.T.2.1	Electro modulation of the neural system	Components for electro-modulation of nerves (heart, retina, etc)	G.T.2.2	*In vivo* electrochemical detection	Invasive or body contacting technologies which are aimed at the diagnosis based on an electrochemical detection/reaction	Y.T.2.3	Various	
D.T.2.1	Osteoconductive nanostructures	Implant nanostructures which provide with structural support and that can allow tissue regeneration	H.T.2.2	Nuclear medicine contract agent (radio-active substance)	Substances for improving visibility in medical imaging based on nuclear medicine	Z.T.2.3	Not sufficiently defined	
E.T.2.1	Dental filling/replacement material	Nanomaterial used for the treatment of dental cavities or tooth defects or cavities	Y.T.2.2	Various				
F.T.2.1	Photothermal therapy	Generation of heat upon light stimulation	Z.T.2.2	Not sufficiently defined				
G.T.2.1	Radiation sensitizer	Generation of heat upon radiation for radio-therapy enhancement	Products with non-pharmacological, immunological, or metabolic action of in vitro diagnostic effect					
H.T.2.1	Nano-sieve	Nanopored structure for filtering (e.g. dialyses membranes)	A.T.2.2v	Fluorescent tracker	Fluorescent substances that attached to substances/structures			

Code	Descriptor	Definition	Code	Descriptor	Definition	Code	Descriptor	Definition
					allowing its detection			
